# Evaluation of the relationship between glycated hemoglobin (HbA1c) levels and diabetes and other diseases: A retrospective study

**DOI:** 10.12669/pjms.42.1.13020

**Published:** 2026-01

**Authors:** Olgun Goktas

**Affiliations:** 1 Dr. Olgun Goktas Associate Professor, Uludag University Family Health Center, 16059, Gorukle Campus-Nilufer, Bursa, Turkey

**Keywords:** Diabetes, Diseases, Family medicine practice, Glycated hemoglobin (HbA1c)

## Abstract

**Objective:**

To retrospectively evaluate the relationships between glycated hemoglobin (HbA1c) levels and diabetes and other diseases in primary care.

**Methodology:**

This study was conducted retrospectively using the available recorded data of individuals registered with the Bursa Uludag University Family Health Center, and who underwent HbA1c measurement during the five years between January 2020 to December 2024. 3520 Visits (N) constituted the study population. Simple random sampling was at least 385 patients with a 5% error rate and a 95% confidence level. 537 (n) Patients who met the inclusion criteria were included in the study sample. The study’s sample power was determined to be 99% and the effect size was determined to be quite high at 0.49.

**Results:**

Of the 537 individuals who participated in the study, 117 (21.8%) were female and 420 (78.2%) were male. The average age of the individuals was determined to be 39.29±14.63. Based on the main findings of the study, allergic disease (β=4.62), gender (β=4.45), anemia (β=3.92), and non-pregnancy status (β=3.22) were found to be multiple associations. Patients with these characteristics are at an estimated risk of having an HbA1c level above 6.5 (R^2^=0.51).

**Conclusion:**

In our study, the male gender, older age, genetic and chronic diseases, allergies, anemia and diabetes were significantly different across HbA1c groups. Allergic diseases, gender, anemia and pregnancy were found to have associations affecting HbA1c levels. Our results regarding HbA1c levels in family medicine are crucial for diagnosis, treatment, follow-up and referral to other clinics.

## INTRODUCTION

The percentage of glycated hemoglobin A1c (% HbA1c) in human whole blood indicates the average plasma glucose concentration over a long period and is used to diagnose diabetes. Common laboratory methods for identifying glycated proteins include high-performance liquid chromatography, immunoassay, and electrophoresis. The accuracy and sensitivity of A1c assays are at least comparable to those of glucose assays. Consequently, the American Diabetes Association, the European Association for the Study of Diabetes and the International Diabetes Federation have concluded that A1c analysis should be considered the primary method for diagnosing diabetes.^1^

The World Health Organization (WHO) recommends an HbA1c level of 6.5% as the cutoff point for diagnosing diabetes, but recommends that a value lower than 6.5% does not exclude diabetes diagnosed using glucose tests.^2^ Besides blood sugar, other factors also influence HbA1c.^3^

The United Nations Political Declaration on Noncommunicable Diseases and the World Health Organization’s NCD Global Action Plan recommend the use of diabetes guidelines and protocols in family medicine practice and the strengthening of countries’ health systems in this regard.^4^ In countries with strong family medicine practices,^5^,^6^ guidelines are more widely used. In Australia, according to guidelines recommended in family medicine clinics, HbA1c testing frequency is six months for those with adequate glycemic control and three months for those with inadequate glycemic control. The American Diabetes Association and the World Health Organization recommend HbA1c testing as the preferred method for diagnosing Type-II diabetes, with a diagnosis of 48 mol/mol (≥6.5). However, HbA1c values may not always be consistent across countries and they have stated that it is appropriate to exclude diabetes at HbA1c values <5.5%. HbA1c can also be used to diagnose prediabetes. It also emphasizes the need to evaluate diabetes symptoms at HbA1c values above 6.5.^7^-^9^ According to the American Diabetes Association’s classification, gestational diabetes is diabetes that becomes apparent in the second or third trimester of pregnancy.^10^ It is emphasized that the HbA1c test should not be used in women younger than two months pregnant.^11^

HbA1c testing is performed in family medicine during general care, routine checkups and diabetes diagnosis and follow-up. While the literature contains many different examples and reports, the relationship between HbA1c and diabetes and certain other diseases is generally examined. However, studies that collectively evaluate its relationships with other diseases and influencing factors are quite limited, creating a gap in the literature. Examining the numerous diseases, including genetic^12^ and chronic diseases^13^, and factors that affect HbA1c levels in the database of a family medicine center with a large population would be beneficial. Evaluating individual data from registered family medicine is crucial in this regard.

## METHODOLOGY

This study aimed to determine the relationship between HbA1c levels and diabetes and other diseases in individuals registered at the family health center and to evaluate the related factors that cause high HbA1c levels. The study was conducted retrospectively using the available recorded data of individuals registered at the Uludag University Family Health Center No. 18 in Bursa, Nilufer. The study retrospectively evaluated patients who underwent HbA1c measurement between 2020 and 2024. The study aimed to investigate which patient characteristics influence HbA1c levels and which characteristics constitute associations for particularly high HbA1c levels. Based on the obtained associations, the risk levels of patients with low or high HbA1c levels were determined based on gender, education, smoking, alcohol consumption, obesity, genetic diseases such as Down Syndrome, hemophilia, cystic fibrosis, etc., cancer, allergic disease, chronic diseases such as chronic obstructive pulmonary disease (COPD), osteoarthritis, hypertension, etc., anemia, pregnancy, diabetes, and age. Early HbA1c measurement in identified and predicted patients at risk is believed to be an early prevention mechanism for diabetes.

The current study has limitations in terms of location, time of implementation, and sample characteristics. The data were collected between 2020 and 2024. The fact that the healthcare institution where the study was conducted was a Family Health Center is limited by the demographic characteristics of the patients who visited the center. It is important to examine the results according to these limitations and characteristics. The high proportion of female patients in the study, the high level of education of the group, the low cigarette and alcohol consumption, and the relatively young average age (39.29±14.63) can be expressed as some of these limitations.

The universe of the study was selected from individuals who applied to the health institution in the relevant period and had their HbA1c level measured. The data obtained in the study revealed that 3520 (N) applications were made to the health center during these five years, and this constituted the study population. It was determined that simple random sampling would be sufficient to include at least 385 patients with a 5% error and a 95% confidence level. In this context, 537 (n) patients who met the inclusion criteria were included in the study sample to provide more accurate results. Furthermore, the study’s sample power was determined to be 99% and the efficacy was quite high at 0.49. As a result of the sample and sample power calculations, it was seen that the study had a sufficient sample size.

### Ethical approval:

The study was performed after approval from the Health Research Ethics Committee of Bursa Uludag University, Faculty of Medicine (Ref. Dated June 11, 2025/ decision no: 2025/702-11/12) following the Declaration of Helsinki.

### Statistical analysis:

Descriptive statistics were presented in the study data analysis using mean, standard deviation, frequency and percentage values. The data were found to be normally distributed because the distribution of the measurements was normal (p>0.05) and the within-group numbers were above normal. An independent samples t-test was used to examine HbA1c groups and age measurements. The chi-square test was used to compare categorical values of patients according to HbA1c groups. Logistic regression analysis was used to determine multiple risk factors (β) affecting HbA1c levels. In the study, p-values below 0.05 were considered statistically significant. Analyses were conducted using SPSS version 25.00.

### Inclusion and exclusion criteria:

Individuals registered at a health center between 2020 and 2024 and who had their HbA1c measurements taken were included in the study. Individuals who migrated or left the region for various reasons, or individuals located outside the region or whose HbA1c measurements were not available were excluded from the study.

## RESULTS

A total of 537 individuals participated in the study. Of the participants, 117 (21.8%) were male and 420 (78.2%) were female. The average age of the individuals was determined to be 39.29±14.63. Of the participants in the study, it was seen that 6.9% of the patients had a high school education level or below, 24.9% were university students, and 68.2% were university graduates. 8.6% of the participants were smokers, and 0.9% were found to drink alcohol. Obesity rates were found to be 30%. Cancer rates were found to be 1.1%, allergic diseases were found to be 15.3% and chronic diseases were found to be 40%. Anemia was found 12.7%, pregnant patients were found to be 15.4% and diabetic patients were found to be 29.4%.

Significant differences were observed in the gender distribution of patients according to HbA1c groups. It was also noted that female participants had a higher HbA1c levels below 6.5 (p=0.01). It was observed that the distribution of patients’ education levels (p=0.64), smoking rates (p=0.92), alcohol consumption rates (p=0.34) and obesity rates (p=0.19) were not significantly different according to HbA1c groups. It was also observed that the rates of patients having genetic diseases were significantly different according to HbA1c groups. It was observed that participants with genetic diseases had a higher rate of HbA1c levels above 6.5 (p=0.04). It was observed that the rates of patients having cancer were not significantly different according to HbA1c groups. The study also showed that participants with and without cancer had HbA1c levels below and above 6.5 at similar rates (p=0.38).

The study showed that the prevalence of allergic diseases (p=0.02), chronic diseases (p=0.01) and anemia (p=0.01) were significantly different in patients according to HbA1c groups. It was also noted that the rates of pregnancy were significantly different in individuals according to HbA1c groups. Pregnant participants were observed to have a higher rate of HbA1c levels below 6.5 (p=0.01). The study also showed that the prevalence of diabetes were significantly different in patients according to HbA1c groups. Participants with diabetes were observed to have a higher rate of HbA1c levels above 6.5 (p=0.04). In the study, we also found that the ages of the patients were significantly different according to HbA1c groups and the average age of the group with HbA1c levels above 6.5 was significantly higher (p=0.01) (Table-I).

**Table-I:** Comparison of general characteristics of patients according to HbA1c groups.

Parameter		HbA1c				Total		p
		<6.5 (n=387)		≥6.5 (n=150)				
		n	%	n	%			
Gender	Male	39	10.1%	78	52.0%	117	21.8%	0.01*
	Female	348	89.9%	72	48.0%	420	78.2%	
Education	Pre-High School	10	2.6%	27	18.0%	37	6.9%	0.64
	University Student	113	29.2%	21	14.0%	134	24.9%	
	University Graduate	264	68.2%	102	68.0%	366	68.2%	
Smoking	No	354	91.5%	137	91.3%	491	91.4%	0.92
	Yes	33	8.5%	13	8.7%	46	8.6%	
Alcohol	No	384	99.2%	148	98.7%	532	99.1%	0.34
	Yes	3	0.8%	2	1.3%	5	0.9%	
Obesity	No	277	71.6%	99	66.0%	376	70.0%	0.19
	Yes	110	28.4%	51	34.0%	161	30.0%	
Genetic Disease	No	380	98.2%	141	94.0%	521	97.0%	0.04*
	Yes	7	1.8%	9	6.0%	16	3.0%	
Cancer	No	386	99.7%	145	96.7%	531	98.9%	0.38
	Yes	1	0.3%	5	3.3%	6	1.1%	
Allergy	No	344	88.9%	111	74.0%	455	84.7%	0.02*
	Yes	43	11.1%	39	26.0%	82	15.3%	
Chronic Disease	No	264	68.2%	58	38.7%	322	60.0%	0.01*
	Yes	123	31.8%	92	61.3%	215	40.0%	
Anemia	No	362	93.5%	107	71.3%	469	87.3%	0.01*
	Yes	25	6.5%	43	28.7%	68	12.7%	
Pregnancy	No	313	80.9%	141	94.0%	454	84.6%	0.01*
	Yes	74	19.1%	9	6.0%	83	15.4%	
Diabetes	No	379	97.9%	0	0.0%	379	70.6%	0.04*
	Yes	8	2.1%	150	100.0%	158	29.4%
Age	*X±SD* (mean±SD)	28.84±6.94		51.01±16.13		39.29±14.63		0.01*

* Significant difference at 0.05 level

To investigate the risk factors affecting HbA1c levels, the results were included in the logistic regression model. Results found to be univariately significant in previous analyses were included in the model. According to the results, allergic disease, gender, anemia and pregnancy were found to be independent risk factors. The model’s success rate was 94%, indicating a high rate. The Nagel-Kerke R^2^ value for this model was calculated as 0.51. Accordingly, the independent variables explained approximately 51% of the variation in the HbA1c variable examined.

When interpreting the significant variables, it was found that patients with allergic disease findings had a 4.62 (95% CIA 1.46-7.88) times higher risk of having an HbA1c level above 6.5 compared to patients without it. Male patients in the study had a 4.45 (95% CIA 2.02-5.21) times higher risk of having an HbA1c level above 6.5 compared to other patients. Patients with anemia have a 3.92 (95% CIA 1.65-6.89) times higher risk of having an HbA1c level above 6.5. Non-pregnant patients have a 3.22 (95% CIA 1,84-5.23) times higher risk of having an HbA1c level above 6.5 compared to other patients. According to the study findings, a preliminary estimate can be made for patients at risk of having an HbA1c level above 6.5 based on allergic disease, gender, anemia and pregnancy status. Pregnancy, as a physiological condition, should be considered as an influencing condition rather than a risk factor, unlike diseases (Table-II).

**Table-II:** Determination of Multiple Risk Factors for Hba1c Levels Above 6.5.

Definitive Diagnosis	W	p	Exp(β)	95% G.A. EXP(β)	
				Bottom	Top
Allergic Disease (+)	9.13	0.01*	4.62	1.46	7.88
Gender (Male)	5.51	0.01*	4.45	2.02	5.21
Anemia (+)	8.85	0.01*	3.92	1.65	6.89
Pregnant (-)	4.63	0.01*	3.22	1.84	5.23

** Logistic Regression Analysis,

* Significant relationship at 0.05 level: Nagelkerke R^2^=0.51

## DISCUSSION

In this study, male gender, genetic and chronic diseases, allergies, anemia, older age and diabetes were significantly different across HbA1c groups. In the logistic regression model, allergic diseases, gender, anemia and pregnancy were identified as independent risk factors affecting HbA1c levels. The results of our study are important for evaluating the factors affecting HbA1c levels in family medicine practice. Family physicians should utilize their diverse clinical experience in addition to existing guidelines and protocols and continue to collaborate with other clinics.

HbA1c is used as a diagnostic test for diabetes in many countries worldwide. According to evidence-based medicine principles, it is associated with decision-making outcomes, particularly by evaluating microvascular complications. HbA1c is advantageous as a diagnostic test, but clinicians should also consider accessibility, cost and other comorbidities during decision-making.^14^ Management of hyperglycemia in Type-II diabetes is also paralleled by HbA1c measurements and weight loss. Following a diabetes diagnosis with HbA1c, achieving early weight loss goals and the magnitude of weight loss were associated with greater reductions in HbA1c levels.^15^-^17^ In our study, individuals with diabetes were found to have higher HbA1c levels above 6.5. However, there was no significant difference in the prevalence of obesity. HbA1c levels were similarly above and below 6.5 in obese and non-obese individuals.

HbA1c is reported to be a good predictor of the lipid profile due to its significant association with cholesterol and triglyceride levels, as well as long-term glycemic control in Type-II diabetic patients.^18^-^21^ It has also been emphasized that HbA1c levels correlate with the mean intima-media thickness of bilateral middle carotid arteries.^22^,^23^ HbA1c levels have also been reported to correlate with the mean duration of diabetes, mean neuropathy disability score and slowing of nerve conduction velocity.^24^ High HbA1c levels are also reported in Type-II diabetic patients with non-fatty liver disease^25^ and anxiety disorders.^26^ In our study, HbA1c levels were generally above 6.5 in those with any chronic disease.

One study found high HbA1c levels to be a cause of poor hypoglycemic control and these levels were also correlated with younger patient age, female gender and chronic diseases.^27^ In contrast, in our study, high HbA1c levels were correlated with chronic diseases, while higher levels were found in older age and male gender. Poor glycemic control has been shown to lead to dangerous maternal and perinatal outcomes in unplanned pregnancies and cesarean deliveries.^28^ Another study in patients with gestational diabetes reported a non-significant difference in HbA1c levels before and after vitamin D treatment.^29^ In our study, pregnant women were found to have HbA1c levels below 6.5 more often than non-pregnant women. The lower HbA1c levels in pregnant women in our study may be due to physiological changes and various factors during pregnancy. Therefore, physiological changes and various factors during pregnancy may make HbA1c less reliable and may require consideration of various factors. One study highlights the influence of genetic factors on long-term HbA1c response,^30^ and another study reports that after three months of follow-up, the decrease in HbA1c with glycemic response was greatest in the variant genotype.^31^ In our study, the prevalence of genetic diseases differed significantly among individuals with HbA1c groups. Those with genetic diseases were more likely to have HbA1c levels above 6.5. One study emphasized that low serum iron and transferrin saturation levels are associated with increased HbA1c values and that iron deficiency should be corrected before HbA1c interpretation.^32^ Another study demonstrated that iron deficiency anemia was associated with increases in HbA1c concentrations and decreases in HbA1c after iron therapy. This emphasizes the importance of considering iron deficiency anemia when making diagnostic or treatment decisions based on HbA1c levels.^33^ In our study, HbA1c levels were significantly higher in patients with anemia. In a sensitivity analysis study defined according to HbA1c levels, it is emphasized that allergic rhinitis (AR) and diabetes mellitus (DM) share a common cause of inflammation and show mutual inverse relationships regardless of gender in individuals aged 30 years and over.^34^ Similarly, in our study, it was determined that HbA1c levels were significantly affected by advanced age and allergic diseases. A randomized controlled systematic review reported that interventions by community health workers in adults with Type-II diabetes resulted in significant reductions in HbA1c levels in low-income ethnic minority groups.^35^

Our study found differences in the factors affecting HbA1c levels in individuals in our region, depending on the situation in secondary and tertiary healthcare settings. Clinical guidelines and protocols, as well as the holistic and consistent approach of the family physician, are crucial in making decisions about different HbA1c test levels. Important conclusions have been drawn regarding which values should be used in normal and abnormal situations. Given that HbA1c levels in the blood can lead to different outcomes and abnormal processes for various reasons, we emphasize the need for family physicians to approach individuals holistically and collaborate with other clinics. There is a need to share the results of HbA1c blood levels in different situations and the various influencing factors in the literature. Our results should not be generalized or interpreted for all patients. However, future extensions of the study to different groups, regions, or types can contribute to the literature.

### Strengths and Limitations:

The strengths of the study are the frequent and practical use of HbA1c in family medicine and the creation of a large database for research. Since our study is retrospective, the possibility that some of the results of consultations at other clinics were not included in our records may have created a limitation. Additionally, individuals who left our region or migrated abroad may have also created limitations.

## CONCLUSION

In our study, the prevalence of male gender, older age, genetic and chronic diseases, allergies, anemia and diabetes were significantly different across HbA1c groups. Allergic diseases, gender, anemia and pregnancy were found to have associations affecting HbA1c levels. Our results regarding HbA1c levels in family medicine are crucial for diagnosis, treatment, follow-up and referral to other clinics.
